# Characterization of a new splicing variant of powdery mildew resistance gene *Pm4* in synthetic hexaploid wheat YAV249

**DOI:** 10.3389/fpls.2022.1048252

**Published:** 2022-10-26

**Authors:** Yuli Jin, Tiantian Gu, Xiuquan Li, Hong Liu, Guohao Han, Zhipeng Shi, Yilin Zhou, Jieru Fan, Jing Wang, Wei Liu, He Zhao, Diaoguo An

**Affiliations:** ^1^ Center for Agricultural Resources Research, Institute of Genetics and Developmental Biology, Chinese Academy of Sciences, Shijiazhuang, Hebei, China; ^2^ Institute of Biotechnology and Food Science, Hebei Academy of Agriculture and Forestry Science/the Key Laboratory of Plant Genetic Engineering of Hebei Province, Shijiazhuang, Hebei, China; ^3^ The National Key Facility for Crop Gene Resources and Genetic Improvement, Institute of Crop Science, Chinese Academy of Agricultural Sciences, Beijing, China; ^4^ The State Key Laboratory for Biology of Plant Disease and Insect Pests, Institute of Plant Protection, Chinese Academy of Agricultural Sciences, Beijing, China; ^5^ The Innovative Academy for Seed Design, Chinese Academy of Sciences, Beijing, China

**Keywords:** *Blumeria graminis* f. sp. *tritici*, *PmYAV*, *Pm4a*, markers, marker-assisted selection

## Abstract

Powdery mildew, caused by *Blumeria graminis* f. sp. *tritici* (*Bgt*), is a destructive fungal disease of wheat throughout the world. Utilization of effective powdery mildew resistance genes and cultivars is considered as the most economic, efficient, and environmental-friendly method to control this disease. Synthetic hexaploid wheat (SHW), which was developed through hybridization of diploid *Aegilops* and tetraploid wheat, is a valuable genetic resource for resistance to powdery mildew. SHW line YAV249 showed high levels of resistance to powdery mildew at both the seedling and adult stages. Genetic analysis indicated that the resistance was controlled by a single dominant gene, temporarily designated *PmYAV*. Bulked segregant analysis with wheat 660K single nucleotide polymorphism (SNP) array scanning and marker analysis showed that *PmYAV* was located on chromosome 2AL and flanked by markers *Xgdm93* and *Xwgrc763*, respectively, with genetic distances of 0.8 cM and 1.2 cM corresponding to a physic interval of 1.89 Mb on the Chinese Spring reference genome sequence v1.0. Sequence alignment analysis demonstrated that the sequence of *PmYAV* was consistent with that of *Pm4a* but generated an extra splicing event. When inoculated with different *Bgt* isolates, *PmYAV* showed a significantly different spectrum from *Pm4a*, hence it might be a new resistant resource for improvement of powdery mildew resistance. The flanked markers GDM93 and WGRC763, and the co-segregated markers BCD1231 and JS717/JS718 were confirmed to be easily performed in marker-assisted selection (MAS) of *PmYAV*. Using MAS strategy, *PmYAV* was transferred into the commercial cultivar Kenong 199 (KN199) and a wheat line YK13 was derived at generation BC_3_F_3_ from the population of YAV249/4*KN199 due to its excellent agronomic traits and resistance to powdery mildew. In conclusion, an alternative splicing variant of *Pm4* was identified in this study, which informed the regulation of *Pm4* gene function.

## Introduction

Powdery mildew, caused by *Blumeria graminis* f. sp. *tritici* (*Bgt*), is a destructive fungal disease of common wheat throughout the world, accounting for approximately 5% of the total yield losses caused by wheat pathogens and pests (Serge et al., 2019). Utilization of effective powdery mildew resistance genes (*Pm* genes) and resistant cultivars is the most economic, efficient, and environmental-friendly method to control this disease.

So far, more than 100 *Pm* genes/alleles from wheat and its relatives have been reported, including formally named *Pm1* to *Pm68* at 63 loci (noting that *Pm8*=*Pm17*, *Pm18*=*Pm1c*, *Pm22=Pm1e*, *Pm23=Pm4c*, and *Pm31*=*Pm21*) and 30 temporarily named genes (He et al., 2020; McIntosh et al., 2020). With the development of sequencing technologies and releases of reference genomes in succession, 13 *Pm* genes, including *Pm1a*, *Pm2*, *Pm3*, *Pm4b*, *Pm5e*, *Pm8*, *Pm17*, *Pm21*, *Pm24*, *Pm38*, *Pm41*, *Pm46* and *Pm60* have been cloned (Krattinger et al., 2009; Brunner et al., 2011; Hurni et al., 2013; Moore et al., 2015; Sánchez-Martín et al., 2016; Singh et al., 2018; Xing et al., 2018; Zou et al., 2018; Li et al., 2020; Lu et al., 2020; Xie et al., 2020; Hewitt et al., 2021; Sánchez-Martín et al., 2021). Because of the large-scale deployment of single resistance genes and the evolution of corresponding pathogens, many resistance genes have lost their effectiveness, such as *Pm1*, *Pm3*, *Pm7* and *Pm8*,either regionally or across the world (Jin et al., 2021). Therefore, it is essential to explore novel resistance genes/alleles of powdery mildew and transfer them into wheat cultivars to enrich the diversity of resistance sources and enhance the durability of disease resistance.

Gene *Pm4* has been one of the most widely used powdery mildew resistance genes and can be found in many cultivars all over the world (Ullah et al., 2018; Jin et al., 2021). Since *Pm4* allele *Pm4a* was first mapped on the long arm of wheat chromosome 2A in Khapli (The et al., 1979), five alleles (*Pm4a-Pm4e*) have been reported at that locus (Ullah et al., 2018). Recently, *Pm4b*, encoded a putative chimeric protein of a serine/threonine kinase with multiple C2 domains and transmembrane regions, has been cloned through Mutant Chromosome Flow Sorting and Sequencing (Sánchez-Martín et al., 2021). *Pm4b* consists of seven exons and contains two alternative transcripts, denoted *Pm4b_V1* and *Pm4b_V2*. Subsequently, three new *Pm4* alleles, tentatively denoted as *Pm4f*, *Pm4g* and *Pm4h* were discovered by the diagnostic functional markers JS717/JS718 (Sánchez-Martín et al., 2021). It is noted that other loci, for example, *Pm1*, *Pm2*, and *Pm5* also have multiple allelic variations because of the long term interactions with the co-evolving pathogen. It is reported that the allelic variations of the documented *Pm* genes played important roles in resistance breeding, for their different spectra of effectiveness to pathogen isolates. Therefore, identification of the allelic variations in detail is important, not only for increasing the diversity of resistance resource, but also for understanding the host-pathogen interaction mechanism and applying in resistance gene pyramiding breeding (Srichumpa et al., 2005; Koller et al., 2018).

Synthetic hexaploid wheat (SHW), artificially created hexaploid wheat, is usually developed from crosses of durum wheat, *Triticum turgidum* ssp. *durum* (2n = 4x = 28, AABB), and *Aegilops tauschii* (2n = 2x = 14, DD), followed by chromosome doubling of the F_1_ hybrids (Kamali et al., 2014). SHW possesses valuable genes for wheat improvement including disease resistance, abiotic-stress tolerance and yield related genes, which can be transferred from tetraploid wheat and *Ae*. *tauschii* to common wheat cultivars as a bridge resource (Trethowan and Mujeeb-Kazi, 2008; Li et al., 2011). Since the late 1980s, the International Maize and Wheat Improvement Center (CIMMYT) has developed more than 1000 SHW lines (Das et al., 2016), these SHW lines are widely used to improve wheat quality, yield, and other important agronomic traits all over the world (Li et al., 2018). Some powdery mildew resistance genes from SHW have been reported. The SHW line “XX 194” was derived from the cross of susceptible cultivar *T. durum* “Moroccos 182” and the selected resistant accession *Ae. squarrosa* “AE 457/78”. Lutz et al. (1995) revealed “XX 194” conferred the same response pattern to *Bgt* isolates as *Pm2*. The SHW line “XX 186” which was produced from a cross between susceptible cultivar *T. durum* “Santa Marta” and *Ae. squarrosa* “BGRC 1458” showed a different response pattern from all other named *Pm* genes, being designated *Pm19* (Lutz et al., 1995). Hu et al. (2001) found that SHW line M81 (68.111/RGB-U//WARD/3/*Ae. tauschii* 452) showed resistance to powdery mildew at both the seedling and adult plant stages. Genetic analyses indicated that the resistance gene originated from *Ae. tauschii* (Hu et al., 2001).

YAV249 is a SHW line that showed effective resistance to powdery mildew in the field over multiple years. To make better use of its *Pm* gene(s), the major objectives of this study were to: (i) determine the inheritance of powdery mildew resistance and identify the *Pm* gene in YAV249; (ii) investigate the relationship between *PmYAV* and *Pm4*; (iii) evaluate diagnostic markers for marker-assisted selection (MAS); (iv) apply *PmYAV* in wheat powdery mildew resistance breeding.

## Materials and methods

### Plant materials

SHW line YAV249 was derived from the cross YAV-2/TEZ//*Ae. squarrosa* 249 (*T. durum* and *Ae. tauschii*) and is highly resistant to powdery mildew at all growth stages. Wheat cultivar Shixin 828 (SX828) is susceptible to the tested *Bgt* isolates and used as the susceptible parent to produce F_2_ and F_2:3_ populations for genetic analysis and mapping of *PmYAV* in this study. Wheat cultivar Mingxian 169, which does not carry any known *Pm* genes, was used as a susceptible control for phenotypic evaluation (Qie et al., 2019). The 47 wheat accessions with known *Pm* genes or gene combinations were tested with 24 different *Bgt* isolates collected from diseased wheat fields in different wheat growing areas in China to make comparison with the *Pm* gene(s) in YAV249 (**Table 1**) (An et al., 2019). Additionally, 192 wheat accessions including cultivars, landraces, advanced lines and introduced cultivars were determined by using the flanked or co-segregated markers of *Pm* gene(s) in YAV249 to validate the applicability for MAS (**Supplementary Table 1**). The susceptible commercial cultivar Kenong 199 (KN199) was used as the recurrent parent to cross and backcross with YAV249 to produce progenies.

**Table 1:** Response spectra of YAV249 and wheat accessions with known powdery mildew (*Pm*) resistance genes to 24 different *Blumeria graminis* f. sp. *tritici* (*Bgt*) isolates at the seedling stage.

Cultivars/lines	*Pm* genes	Resistance/susceptible ratio	*Blumeria graminis* f. sp. *tritici* isolates																							
			E01	E05	E06	E07	E09	E11	E13	E15	E16	E17	E18	E20	E21	E23-(1)	E23-(2)	E26	E30-(1)	E30-(2)	E31	E32	E49	E50	E60	E69
YAV249	*PmYAV*	16/8	0	0	0	4	0;	2	1	4	2	0	4	4	2	0	0	0	0	4	4	4	0	4	2	2
Khapli/8cc	*Pm4a*	14/10	2	0	0;	4	0;	0;	0	4	0;	0	4	4	0;	0;	0;	0	0	4	4	4	0	4	3	3
Armada	*Pm4b*	18/6	2	0;	0;	3	0;	0;	0	0;	0;	0;	0	3	0	0	0;	0;	0	4	4	3	0	4	2	0;
81-7241	*Pm23=Pm4c*	23/1	0;	0;	0;	0	0;	0;	0	0;	0;	0;	0	0;	0;	0;	0;	0	0;	3	0;	0;	0	0	0;	0;
Axminster/8cc	*Pm1a*	2/22	4	4	4	3	4	4	0;	4	4	4	4	4	4	2	4	3	4	4	4	4	4	4	4	4
Ulka/8cc	*Pm2*	18/6	0;	0;	0;	0;	0;	0;	1	0;	0	0;	4	4	0;	0;	4	0;	0;	4	0;	0;	0;	4	0;	4
Asosan/8cc	*Pm3a*	5/19	4	4	3	3	4	3	3	0	4	1	3	4	3	4	4	4	0;	2	4	0;	4	4	4	4
Chul/8cc	*Pm3b*	4/20	4	4	0;	3	4	3	0;	0	4	4	4	4	3	3	4	4	3	4	0;	4	4	3	3	3
Sonora/8cc	*Pm3c*	1/23	4	4	4	4	4	4	4	0	3	4	3	4	3	3	4	4	3	4	4	3	4	4	4	4
Kolibri	*Pm3d*	17/7	0	0;	0;	4	0;	3	1	0;	4	0	0	0	0	0;	0;	0	0;	4	0;	0;	4	3	2	3
Mich.Amber/8cc	*Pm3f*	0/24	4	4	4	4	3	4	4	4	4	4	4	4	3	4	4	4	4	4	4	4	4	4	4	4
W150	*Pm3e*	0/24	4	3	4	4	4	4	4	4	4	4	4	4	4	4	4	4	4	4	4	3	4	4	4	4
Hope/8cc	*Pm5a*	0/24	4	4	4	4	4	3	4	4	4	4	4	3	4	3	3	4	3	4	3	3	4	4	3	3
Aquila	*Pm5b*	20/3	0;	0;	0;	0	0;	0;	0	0	0;	0;	4	3	0	0;	0;	0	0;	–	0;	0;	1	4	2	1
Fuzhuang 30	*Pm5e*	15/9	2	1	3	4	0;	3	4	0;	0;	4	1	3	1	3	4	3	1	2	0;	0;	2	1	2	1
Timgalen	*Pm6*	0/24	4	4	4	4	4	4	4	4	4	4	4	4	3	3	3	3	4	4	4	4	3	4	4	4
Coker 747	*Pm6*	4/20	4	4	3	3	3	3	4	4	2	4	4	3	3	3	0;	4	2	4	3	2	3	4	3	4
CI14189	*Pm7*	0/24	4	4	4	4	4	3	4	4	4	4	4	4	4	4	4	4	4	3	4	3	4	4	4	4
Kavkaz	*Pm8*	1/23	4	3	4	4	4	3	3	4	4	4	4	4	3	3	3	3	3	3	4	4	3	4	0	4
Wembley	*Pm12*	24/0	0	0	0	0	0	0	0	0	0	0	0	0	0	0	0	0	0	0	0	0	0	0	0	0
R4A	*Pm13*	22/2	0;	0;	0;	0	0;	0;	0	0;	0;	0	2	1	0	3	0;	0	0;	3	0;	0;	0	0;	1	0;
Brigand	*Pm16*	24/0	0	0;	0	0	0;	0	0	0	0;	0	1	0;	0;	0	0;	0	0	0	0;	0	0	0;	0	0
Amigo	*Pm17*	10/14	4	0;	1	4	3	0;	0	0	0;	2	3	4	0;	0;	0;	4	3	4	4	3	4	4	3	4
MIN	*Pm18=Pm1c*	24/0	0	0;	0;	0	0;	0;	0;	0;	0;	0;	0;	0;	0;	0;	0;	0;	0;	0;	0;	0;	0	0	0	0;
XX186	*Pm19*	0/24	4	4	4	4	4	3	4	4	4	4	3	4	4	4	4	4	4	4	4	4	4	4	4	4
TAM104/Thatcher	*Pm20*	17/7	4	0;	0	0	3	0;	0	0	3	0	2	2	2	4	0;	0	0;	2	4	4	3	0;	0	0;
Nannong 9918	*Pm21*	24/0	0;	0;	0;	0	0;	0;	0;	0;	0;	0;	0	0;	0;	0;	0;	0	0	0;	0;	0;	0	0;	0;	0;
Virest	*Pm22=Pm1e*	22/2	3	0	0	0	0;	0;	0	0;	0;	0	0	0	0	0	0;	0	0;	3	0;	0;	0	0	0	0
Chiyacao	*Pm24*	22/2	0	2	0;	1	0;	0;	0;	4	0;	0;	0;	0;	0	0;	0;	3	0;	0;	0;	0;	0	0;	0;	0;
NCA5	*Pm25*	20/4	0	0	0;	0	0;	0;	1	0;	0;	3	3	0;	0	0;	0;	0	0;	3	2	0;	0	3	0;	0;
5P27	*Pm30*	17/7	2	0;	0;	4	0;	0;	0;	0;	0;	0;	3	4	0;	0;	0;	0	0;	4	3	3	0;	4	0;	0;
NCA7	*Pm34*	14/10	0	2	2	0;	3	0;	3	2	2	0	2	3	3	3	2	4	0;	3	4	0;	3	0	0;	3
NCA3	*Pm35*	20/4	0;	0	3	3	1	0;	0;	1	0;	0;	0;	1	0;	3	0;	0;	0;	0;	3	0;	0;	0	0;	0;
Pm36	*Pm36*	18/6	0;	0;	0;	4	0;	0;	0;	0;	0;	0;	4	3	0	0;	0;	0;	0;	3	0;	3	0;	4	2	0;
GRY19	*Pm40*	16/8	3	1	0;	4	0;	0;	0;	0;	0;	0;	4	4	0;	0;	0;	0;	0;	4	4	4	0;	4	2	0;
Tabasco	*Pm46*	24/0	0	0	0	0	0	0;	0	0	0;	0;	1	0	0	0;	0;	0	0;	2	0;	0;	0	0;	0;	0;
CH7086	*Pm51*	21/3	0;	0;	0;	0;	0;	0;	0	0;	0;	0;	3	0;	1	0;	0;	0;	0	4	0;	0;	0	4	0;	2
Normandie	*Pm1+2+9*	3/21	4	3	3	4	3	3	3	3	0;	4	3	4	3	3	4	4	2	4	4	4	4	0;	3	4
Maris Huntsman	*Pm2+6*	21/3	0	0;	0	0;	0;	0;	0	0;	0;	0	3	3	0	0;	0;	0	1	3	0;	0;	0	1	0	0;
Maris Dove	*Pm2+Mld*	22/2	0;	0	0	0	0;	0;	0	0	0;	0	0	4	0	0;	0;	0	0;	4	0;	0;	0	0	0	0;
Mission	*Pm4b+5b*	18/6	3	0;	0;	4	0;	0;	0;	0;	0;	0;	0;	4	0;	0;	0;	0	0;	4	3	4	0;	0;	0;	0;
Bianmian 3	*Pm4+8*	17/7	2	0;	0;	4	0;	0;	0;	0;	0;	1	4	4	0;	0;	0;	0	0;	3	4	4	0;	3	0;	0;
Coker 983	*Pm5+6*	3/21	4	4	3	3	3	3	3	3	3	4	4	3	4	0;	2	4	3	4	4	2	3	3	3	3
Xiaobaidongmai	*Pm*”*XBD*”	15/9	0;	2	3	3	0;	4	3	0;	0;	3	0;	0;	0;	4	4	0;	0;	0;	4	0;	1	1	0;	4
NCV4	*Non-Pm1*	13/11	4	2	0	2	3	0;	0	2	3	3	4	3	3	0;	3	4	0;	0	2	0;	3	2	3	2
Chancellor	–	0/24	4	4	3	4	4	4	4	4	4	4	4	4	4	4	4	4	4	4	4	4	4	4	4	4
Funo	–	0/24	4	4	4	4	4	4	4	3	4	4	4	4	4	4	4	3	4	4	4	4	4	3	4	4
Era	–	15/9	1	0;	0	0;	0;	4	0;	0;	3	0;	4	4	0;	3	0;	0	0;	4	0;	4	0;	4	0;	4

Infection types 0-2 were resistant and 3-4 were susceptible as described by Sheng, 1988; Wang et al., 2005.

### Reaction-phenotyping to *Bgt* isolates

At the adult stage, YAV249 was inoculated with *Bgt* isolates mixture of E09, E11, E18 and E20 in the field nurseries with three replicates. The assessments were performed from 2019 to 2021 at Luancheng Agro-Ecological Experimental Station (37° 53′ 15″ N, 114° 40′ 47″ E), Chinese Academy of Sciences, Shijiazhuang, Hebei Province, China. YAV249 was planted with four rows, 30 seeds per row (1.5 m), and wheat cultivar Mingxian 169 was planted around the plot as susceptible control for each replicate. When Mingxian 169 showed severe disease symptoms, the phenotype reaction of YAV249 was assessed at least two times in weekly intervals with a 0-9 scale for ITs, ITs 0-2 were considered as highly resistant, ITs 3-4 as moderately resistant, ITs 5-6 as moderately susceptible and ITs 7-9 as highly susceptible (Sheng and Duan, 1991).At the seedling stage, YAV249 and 47 wheat accessions with known *Pm* genes or gene combinations were tested for response to 24 single-pustule-derived *Bgt* isolates to compare their resistance spectra (**Table 1**). *Bgt* isolate E09 prevalent in the main wheat producing regions of China (Zhou et al., 2005) was used to inoculate YAV249, SX828, KN199 and progenies of YAV249/SX828 and YAV249/KN199. At least 20 plants of each variety/progeny were used for phenotyping of *Bgt* isolates in three replicates. Response to powdery mildew of all materials was executed in a greenhouse. Materials were planted in the rectangular trays with 128 wells and inoculated at the one-leaf stage by dusting fresh conidia of a *Bgt* isolate, and Mingxian 169 was planted randomly in the trays as the susceptible control. After inoculation, the trays were treated in a high humidity environment at 22 ± 2°C and 10 h of darkness at 18°C. When the first leaf of Mingxian 169 showed full development of pustules about 14-15 days after inoculation, infection types (ITs) for each plant were scored using a 0-4 scale, and plants with ITs 0-2 were regarded as resistant and those with ITs 3 and 4 susceptible (Sheng, 1988; Wang et al., 2005).

### Microscopic analyses of powdery mildew resistance reaction

Microscopic analyses of the plants were performed as previously described (Wang et al., 2014). Two cm leaf segments of YAV249 and SX828 at 7 days post-inoculation (dpi) inoculated with *Bgt* isolate E09 were fixed at 37°C for 24 h in 2 ml of Carnoy’s Fluid (ethanol: acetic acid, 3:1, v/v), then stained with 2 ml of 0.6% (w/v) Coomassie blue solution for 3 min. Excess dye was rinsed off carefully with distilled water. Samples were observed under an Olympus BX-53 microscope (Olympus, Tokyo, Japan).

### Bulked segregant analysis with the wheat 660K SNP array

Genomic DNA was extracted from young leaf tissues after evaluation of their powdery mildew reactions following the method of cetyletrimethylammonium bromide (CTAB) (Sharp et al., 1988). Resistant and susceptible bulks were made from equal amounts of DNA from 20 homozygous resistant and 20 homozygous susceptible F_2:3_ families of YAV249/SX828 for Illumina wheat 660K single nucleotide polymorphism (SNP) array scanning by China Golden Marker Company (Beijing, China). Single nucleotide polymorphism genotype calling and clustering was performed with software Genome Studio Polyploid Clustering v1.0 (Illumina, http://www.illumina.com). The monomorphic and poor quality SNP markers were excluded from further analyses. Then, the distribution of polymorphic SNPs between the resistant and susceptible DNA bulks on wheat chromosomes was analyzed. An enriched peak of differential SNPs was considered as the candidate interval of the *Pm* gene(s) in YAV249.

### Molecular markers analysis

Based on the predicted interval of 660K SNP array scanning, 52 documented SSR molecular markers in the interval, including 40 SSR markers published on the GrainGenes website (http://wheat.pw.usda.gov) and 12 SSR markers developed by Ullah et al. (2018), were used to test the polymorphisms between the resistant and susceptible parents and bulks. The polymorphic markers between the parents and the bulks were used to genotype the F_2:3_ families of YAV249/SX828. Then, the markers were aligned on the Chinese Spring reference genome sequence v1.0 (International Wheat Genome Sequencing Consortium (IWGSC), 2018) to confirm their physical locations.

PCR procedure was performed as previously described (Ma et al., 2015). The amplification products of marker BCD1231 were detected by agarose gel electrophoresis with a concentration of 1.5%, and stained with Super GelRed, while the remaining amplification products were separated in 8% non-denaturing polyacrylamide gels with 1×TBE buffer, and visualized by silver staining (Santos et al., 1993).

### Map construction

A Chi squared (χ^2^) test was carried out to investigate deviations of the observed phenotypic data of the F_2:3_ families from the theoretically expected segregation ratios for analyzing the goodness-of-fit. Then, the linkage map of *PmYAV* was constructed using MAPMAKER 3.0 and the Kosambi function as reported previously (Kosambi, 1944; Lander et al., 1987).

### Cloning and analysis of the *Pm4* homologous sequence in YAV249

Since *PmYAV* was mapped to the *Pm4* interval, the homologous sequence of YAV249 was cloned and assembled by using a homology-based cloning strategy according to the previous report on cloning *Pm4* (Sánchez-Martín et al., 2021). Then, the homologous sequence of YAV249 was compared with the cloned *Pm4* sequence.

### RNA extraction and gene expression analysis

The wheat leaf samples of YAV249 were collected at 0, 2, 4, 8, 12, 24, 48 and 72 hours post inoculation (hpi) after inoculating *Bgt* isolate E09 and with three biological replicates. Total RNA was extracted using RNAiso Plus (TaKaRa, Shiga, Japan). For each sample, 2 μg of RNA was used for reverse transcription with a FastQuant RT Kit (Tiangen, Beijing, China). The transcript levels were detected by qRT-PCR on Bio-Rad CFX 96 with TB Green Premix Ex Taq™ II (TaKaRa, Shiga, Japan). The procedure included an initial step at 95°C for 30 s followed by 40 cycles of 95°C for 15 s, 60°C for 30 s, and 72°C for 10 s. The expression levels of target genes were normalized to that of *TaActin*. All qRT-PCR assays were performed in three independent replications.

### Evaluation of the markers for MAS

The 192 wheat accessions including cultivars, landraces, advanced lines and introduced cultivars were tested by using the flanked or co-segregated markers of *PmYAV* and a specific marker of *Pm4* to evaluate their applicability for MAS breeding (**Supplementary Table 1**). If the polymorphic band(s) of a marker were same in both YAV249 and the tested accessions, it was considered not suitable for MAS. However, when the markers that amplified products in YAV249 that were different from the products in the tested accessions, this indicated that the marker could be used to detect *PmYAV* when it was transferred into these accessions and considered to be applicable for MAS breeding.

### Application of *PmYAV* in wheat powdery mildew breeding

To transfer *PmYAV* into susceptible wheat cultivars to improve the resistance to powdery mildew, YAV249 was crossed and backcrossed with wheat cultivar KN199 that is widely grown in Huanghuai region (Zhang et al., 2011). Combined with identification of powdery mildew resistance and evaluation of agronomic traits, the flanked markers and co-segregated marker were used to trace *PmYAV* in the BC_1_F_1_, BC_2_F_1_, BC_3_F_1_, BC_3_F_2_ and BC_3_F_3_ populations.

The homozygous resistant progeny line YK13 derived from BC_3_F_3_ generation was selected to evaluate agronomic performance along with the parents YAV249 and KN199. These materials were planted at Luancheng Agro-ecosystem Experimental Station (37° 53′ 15″ N, 114° 40′ 47″ E) from 2019 to 2021 with three replicates. Each line was planted in five rows (1.5 m length and 0.25 m between rows) and 20 seeds per row. For each parent/line, 10 plants in the middle three internal rows were randomly sampled to investigate the plant height (PH), spike numbers per plant (SNPP), spike length (SL), spikelet numbers per spike (SNS), sterile spikelet numbers per spike (SSNS), kernel numbers per spike (KNS), thousand-kernel weight (TKW) and kernel related traits. PH and SNPP were assessed based on the mean of 10 plants. SL, SNS, SSNS and KNS were identified based on the mean of the main spike of 10 plants. TKW and kernel related traits (kernel length (KL), kernel width (KW), kernel length/kernel width (KL/W)) of at least 500 kernels randomly were measured three times using the rapid SC-G grain appearance quality image analysis system (WSeen Detection, Hangzhou, China). Analysis of variance (ANOVA) and least significant difference (LSD) test were performed with SPSS statistics v20.0 software (SPSS, Chicago, USA) to test the significance of differences between YK13 and its parents YAV249 and KN199 for each agronomic trait.

## Results

### Inheritance of powdery mildew resistance in YAV249

**Figure 1:**
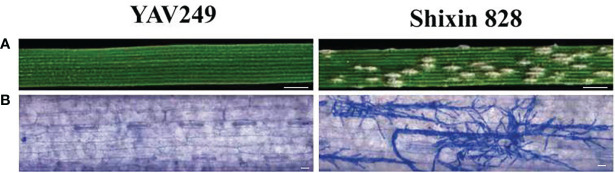
Macroscopic and microscopic characterization of YAV249 and Shixin 828 after inoculating isolate E09 of *Blumeria graminis* (f) sp. *tritici* (*Bgt*). **(A)** Macroscopic view of the infected representative leaf segments from YAV249 and Shixin 828 at 10 d post-inoculation (dpi). Bar, 5 mm. **(B)** Microscopic view of leaves from YAV249 and Shixin 828 at 7 dpi to visualize fungal mycelia. Bar, 100 μm.

From 2019 to 2021, YAV249 was highly resistant with ITs 0-1 to the *Bgt* isolate mixture of E09, E11, E18 and E20 at the adult stage. When inoculated with *Bgt* isolate E09 at the seedling stage, YAV249 was highly resistant with IT 0, while SX828 was highly susceptible with IT 4 (**Figure 1A**). The results of microscopic observation indicated that many mycelia were produced in SX828 but not in YAV249 (**Figure 1B**). Twenty F_1_ plants of the cross YAV249/SX828 were resistant with IT 0-1, indicating that the resistance of YAV249 to *Bgt* isolate E09 was controlled by dominant *Pm* gene(s). The F_2_ population from the cross of YAV249/SX828 segregated with 231 resistant and 88 susceptible plants, which fit the theoretical ratio of 3:1 for monogenic segregation of a dominant gene (*χ^2^* = 1.14; *P* = 0.29). All 319 F_2_ plants were transplanted in the field to produce F_2:3_ families. The 250 F_2:3_ families segregated with 64 homozygous resistant (RR), 119 segregating (Rr) and 67 homozygous susceptible (rr). The result further confirmed the ratio of monogenic inheritance of a single dominant powdery mildew resistance gene (*χ^2^* = 0.65; *P* = 0.72). Therefore, it was concluded that the resistance to *Bgt* isolate E09 in YAV249 was controlled by a single dominant gene, tentatively designated *PmYAV*.

### Bulked segregant analysis with wheat 660K SNP array

**Figure 2:**
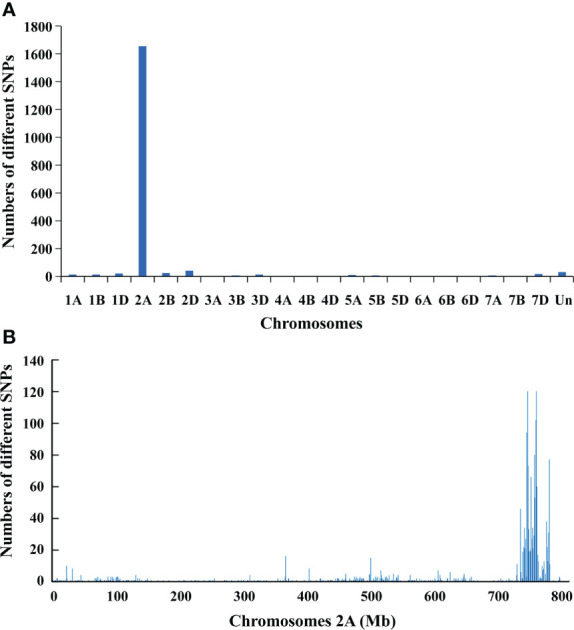
**(A)** Distribution of polymorphic SNP loci between the resistant and susceptible DNA bulks from the F_2:3_ families of YAV249/Shixin 828 on 21 chromosomes. **(B)** Density of polymorphism SNP loci on chromosome 2A with 1 Mb intervals.

To map *PmYAV*, the resistant and susceptible DNA bulks were genotyped with the 660K SNP array. A total of 1,901 SNPs which was distributed across 21 wheat chromosomes were homozygous polymorphic between the two bulks. Among them, 1,653 SNPs were enriched on chromosome 2A, and 248 SNPs were distributed on other chromosomes (**Figure 2A** and **Supplementary Table 2**). Of the 1,653 SNPs located on chromosome 2A, 1,179 SNPs were enriched in an interval of 29 Mb (734 Mb to 763 Mb) on chromosome 2AL (**Figure 2B**), indicating that *PmYAV* was mapped in or near this region.

### Molecular mapping of *PmYAV*

**Figure 3:**
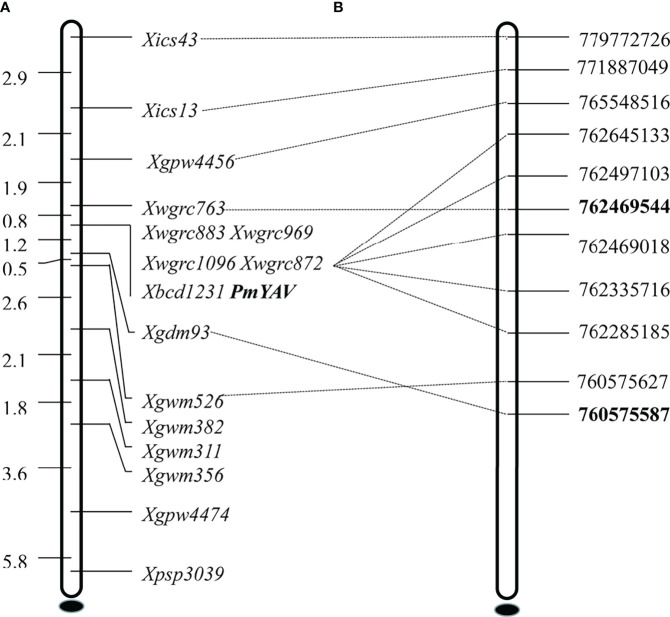
Genetic linkage map **(A)** and physical map **(B)** of powdery mildew resistance gene *PmYAV* using the F_2:3_ families of YAV249/Shixin 828.

Based on the mapping interval of the 660K SNP array, 52 SSR molecular markers located on chromosome 2AL were used to screen between resistant parent YAV249, susceptible parent SX828, resistant bulk and susceptible bulk. Among them, 14 markers PSP3039, GPW4474, GWM356, GWM311, GWM382, GDM93, GWM526, GPW4456, BCD1231, WGRC763, WGRC872, WGRC1096, WGRC869 and WGRC883 showed consistent polymorphism between parents and bulks. Subsequently, these markers were used to genotype the 250 F_2:3_ families and confirm linkage with *PmYAV*. The result demonstrated that *PmYAV* co-segregated with five markers *Xwgrc883*, *Xwgrc969*, *Xwgrc1096*, *Xwgrc872* and *Xbcd1231* and was flanked by *Xwgrc763* and *Xgdm93* with genetic distances 0.8 cM and 1.2 cM corresponding to a physic interval of 1.89 Mb (760.58-762.47 Mb) on the Chinese Spring reference genome sequence v1.0 (**Figure 3**). There were four reported and formally designated *Pm* genes including *Pm4*, *Pm33*, *Pm50* and *Pm65* on chromosome 2AL (Li et al., 2019), and *PmYAV* mapped closed to *Pm4*.

### Cloning and analysis of the *Pm4* homologous sequence in YAV249

**Figure 4:**
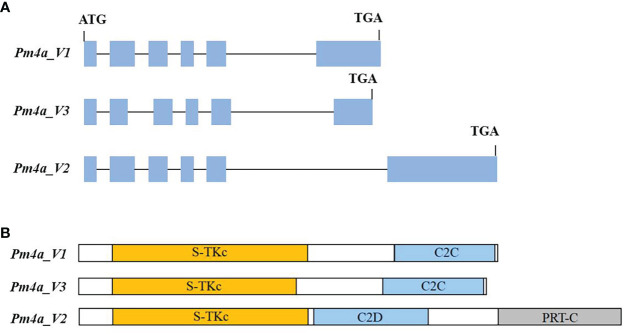
**(A)** Schematic representation of gene structures of *PmYAV* splicing variants. Gene exons and introns are shown by blue boxes and black lines, respectively. **(B)** *Pm4a_V1*, *Pm4a_V2* and *Pm4a_V3* protein isoforms with domains indicated by colours: yellow, serine/threonine kinase (S-TKc); light blue, C2; grey, phosphoribosyl transferase C-terminal (PRT-C).

To further explore the relationship between *PmYAV* and *Pm4*, we genotyped all the 250 F_2:3_ families from YAV249/SX828 with the functional molecular marker JS717/JS718 of *Pm4*. JS717/JS718 showed positive amplification in YAV249 and co-segregated with *PmYAV* in the F_2:3_ families. Then, the *Pm4* homologous coding sequence in YAV249 was cloned using nested PCR, and the result showed that *Pm4* homologous sequence in YAV249 was totally consistent with the *Pm4a* sequence. However, *PmYAV* generated an extra splicing event compared to the previously reported *Pm4a_V1* and *Pm4a_V2* (Sánchez-Martín et al., 2021), suggesting that there was an extra 67 bp-intron in *PmYAV*. The intron was located on the second exon in the serine/threonine kinase domain of *Pm4a_V1* (**Figure 4**).

### Gene expression analysis of *PmYAV*

**Figure 5:**
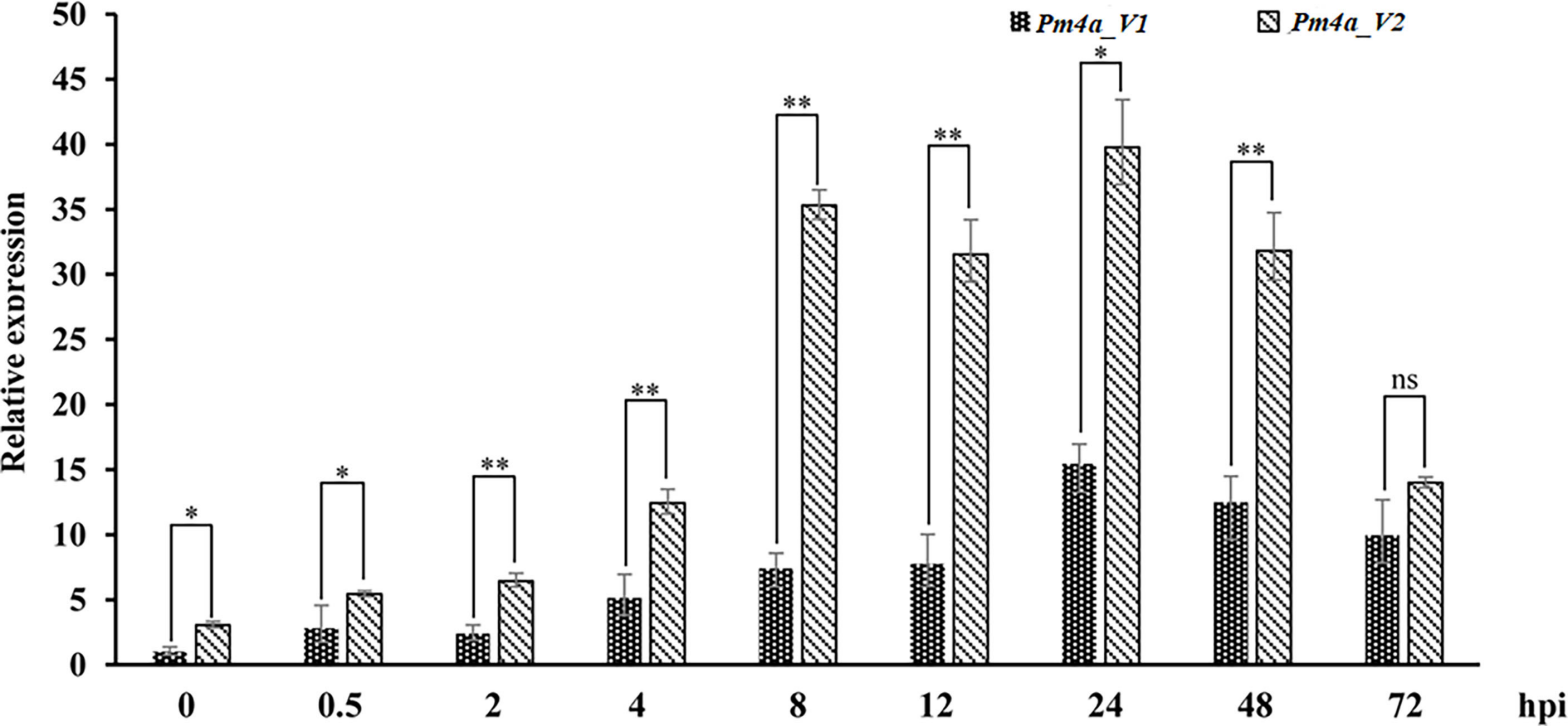
Expression patterns of the *Pm4a_V1* and *Pm4a_V2* splice variants in YAV249 during a 72-hour time course. Error bars represent SD based on three independent repeats. Asterisks indicate significant differences (*t*-tests) between *Pm4a_V1* and *Pm4a_V2* at each time point (*P < 0.05, **P < 0.01, ns: not significant). *TaActin* was used as the internal control.

To investigate the expression patterns of *PmYAV*, we performed qRT-PCR analysis. *Pm4a_V1* and *Pm4a_V2* in YAV249 showed similar expression patterns and were upregulated after *Bgt* isolate E09 infection, peaked at 24 hpi, and then reduced at later stages between 48 and 72 hpi. The level of *Pm4a_V2* was higher significantly than that of *Pm4a_V1* at most of the time points (**Figure 5**). We speculated that *Pm4a_V2* had a greater contribution than *Pm4a_V1* to resistance in YAV249.

### The difference of powdery mildew resistance spectrum between YAV249 and documented *Pm4a* stock

When inoculated with 24 *Bgt* isolates, *Pm4a* was resistant to 14 out of 24 *Bgt* isolates, including E01, E05, E06, E09, E11, E13, E16, E17, E21, E23-1, E23-2, E26, E30-1 and E49, while *PmYAV* was also resistant to E60 and E69 besides those 14 *Bgt* isolates (**Table 1**). Therefore, *PmYAV* showed a different reaction to two *Bgt* isolates compared to *Pm4a*.

### Evaluation of the markers of *PmYAV* for MAS

**Figure 6:**
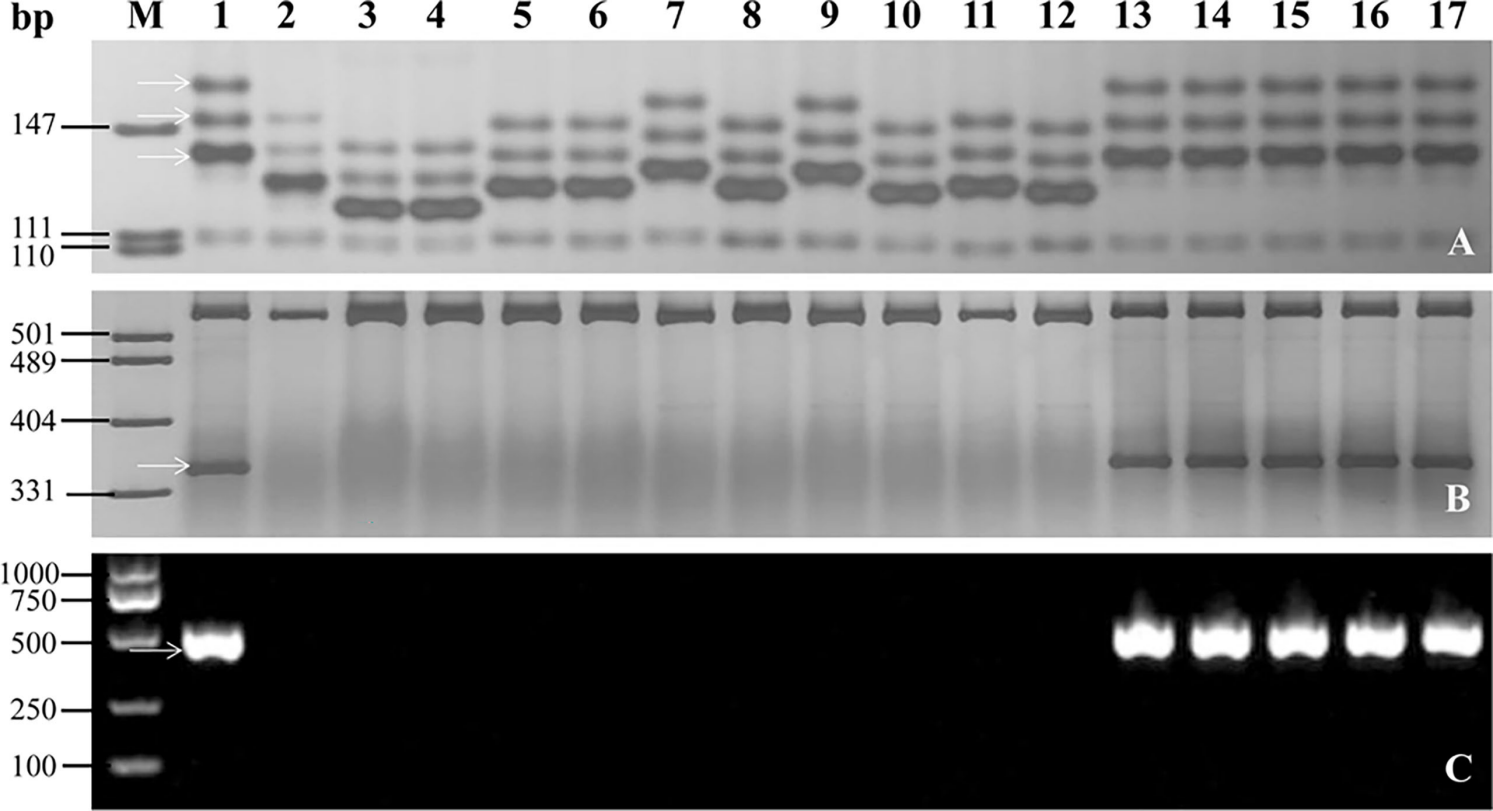
Amplification patterns of the flanked markers GDM93 **(A)** and WGRC763 **(B)** and the co-segregated marker BCD1231 **(C)** of *PmYAV* in YAV249, Shixin 828 and selected wheat cultivars/lines. M: pUC19/*Msp*I; 1: YAV249; 2: Shixin 828; 3: Shi 4185; 4: Shiyou 17; 5: Shimai 18; 6: Shixin 633; 7: Ji 5265; 8: Kenong 199; 9: Zhengyumai 9989; 10: Zhou 16; 11: Jinmai 41; 12: Gaoyou 503; 13: Xinmai 1998; 14: ROANE; 15: Safha 3; 16: Aikang 58; 17: Taixue 12. The white arrows indicate the specific amplified fragments.

To investigate the applicability of the markers linked or co-segregated with *PmYAV* in MAS, the flanked markers GDM93 and WGRC763 and the co-segregating markers BCD1231 and JS717/JS718 of *PmYAV* were used to test 192 wheat accessions (**Supplementary Table 1**). Markers GDM93 and WGRC763 amplified specific bands that differed from YAV249 in 147, and 140 out of 192 accessions respectively. The same as JS717/JS718, BCD1231 amplified different bands between YAV249 and 137 out of 192 accessions. The results indicated that these four markers could be used in MAS for detecting *PmYAV* in those wheat genetic backgrounds (**Figure 6** and **Supplementary Table 1**). However, this was not the case for all the marker alleles in wheat cultivars Aikang 58, Xinmai 1998, ROANE, Safha 3 and Taixue 12 due to the same bands between YAV249 and them (**Figure 6** and **Supplementary Table 1**).

### Application of *PmYAV* in wheat powdery mildew breeding

To develop a resistance pre-breeding resource, we transferred *PmYAV* into the commercial susceptible cultivar KN199 by MAS, combined with evaluation of powdery mildew resistance and analysis of agronomic traits. An advanced breeding line YK13 was selected from a BC_3_F_3_ population of YAV249/4*KN199 due to its desirable agronomics traits and resistance to powdery mildew. During 2019-2021, analysis of agronomic traits indicated that the *PmYAV* donor parent YAV249 had a greater SL, TKW and KL but a poorer PH than the recurrent parent KN199. Compared with KN199, YK13 showed improved SL, TKW and KL in one or two years. Moreover, there was no significant difference on PH, SNPP, SNS, KNS and KW between YK13 and its recurrent parent KN199 (**Table 2**).

**Table 2:** The agronomic trait evaluation of the advanced breeding line YK13 with its donor parent YAV249 and recurrent parent Kenong 199.

Year	Cultivars/Lines	PH (cm)	SL (cm)	SNPP	SNS	KNS	TKW (g)	KL (mm)	KW (mm)
2019-2020	YAV249	92.2 ± 1.7^a^	13.0 ± 0.2^a^	16.6 ± 1.1^a^	19.0 ± 0.5^a^	49.0 ± 3.1^a^	38.4 ± 1.2^a^	8.2 ± 0.1^a^	3.0 ± 0.1^a^
	Kenong 199	73.2 ± 1.5^b^	8.0 ± 0.1^c^	18.4 ± 1.2^a^	21.0 ± 1.1^a^	46.0 ± 2.7^a^	37.2 ± 2.0^a^	5.9 ± 0.1^c^	3.3 ± 0.1^a^
	YK13	73.8 ± 1.8^b^	9.0 ± 0.1^b^	17.6 ± 0.8^a^	20.6 ± 1.5^a^	52.0 ± 3.5^a^	37.9 ± 1.8^a^	6.7 ± 0.1^b^	3.2 ± 0.1^a^
2020-2021	YAV249	94.0 ± 1.4^a^	12.7 ± 0.3^a^	12.5 ± 0.7^a^	19.5 ± 0.7^a^	65.5 ± 7.1^a^	53.9 ± 3.0^a^	8.6 ± 0.1^a^	3.4 ± 0.1^a^
	Kenong 199	75.8 ± 1.5^b^	8.1 ± 0.3^c^	10.8 ± 1.3^a^	20.8 ± 1.0^a^	63.5 ± 0.7^a^	43.0 ± 2.1^c^	6.0 ± 0.1^c^	3.5 ± 0.1^a^
	YK13	75.3 ± 1.9^b^	8.8 ± 0.5^b^	10.8 ± 1.7^a^	21.5 ± 1.7^a^	77.0 ± 11.7^a^	48.5 ± 2.0^b^	6.8 ± 0.1^b^	3.5 ± 0.1^a^

Values with the same letters in the same column were not significantly different at the P < 0.05 according to the least significant difference test. PH, plant height; SL, spike length; SNPP, spike numbers per plant; SNS, spikelet numbers per spike; KNS, kernel numbers per spike; TKW, thousand-kernel weight; KL, kernel length; KW: kernel width.

## Discussion

SHW lines possess important resistance genes effective against various diseases, therefore, regular screening of SHW genotypes is important for identifying new sources of resistance to powdery mildew that could eventually be used in wheat disease resistance breeding programs. In this study, the SHW line YAV249 derived from the cross YAV-2/TEZ//*Ae. squarrosa* 249 was identified to be resistant to powdery mildew at all stages. Genetic analysis indicated that its resistance was conferred by a single dominant gene, temporarily designated *PmYAV*. Bulked segregant analysis by using wheat 660K SNP array scanning and marker analysis showed that *PmYAV* was mapped in the *Pm4* interval on chromosome 2AL.

Up to now, eight alleles (*Pm4a*-*Pm4h*) have been reported at the *Pm4* locus (Sánchez-Martín et al., 2021), which makes it multiple allelic loci as previously known *Pm1* (Hsam et al., 1998), *Pm2* (Jin et al., 2018), *Pm3* (Bhullar et al., 2010) and *Pm5* (Xie et al., 2020) loci. *Pm4a* was derived from tetraploid wheat (*T. turgidum*) cultivars Yuma and Khapli (Briggle, 1966). However, *Pm4b* was originally reported in three bread wheat cultivars Weihenstephan M1, TP29 and ELS (Wolfe, 1965; The et al., 1979). Subsequently, *Pm4b* was postulated in French cultivar VPM1 (Bariana and McIntosh, 1994). Three alleles at *Pm4* locus, including *Pm4c* (*Pm4c* = *Pm23*), *Pm4d*, and *Pm4e*, were identified in wheat line 81-7241, Tm27d2, and landrace D29, respectively (Hao et al., 2008; Schmolke et al., 2012; Li et al., 2017). *Pm4f*, *Pm4g* and *Pm4h* which were susceptible to most of the tested *Bgt* isolates were discovered by using the haplotype-specific marker JS717/JS718 in a global wheat collection of 512 accessions (Sánchez-Martín et al., 2021). In this study, *PmYAV* was resistant to 16 of 24 *Bgt* isolates at the seedling stage which was different from the tested *Pm4a* and *Pm4b* (**Table 1**). Meanwhile, the donor of *PmYAV* was also different from those of *Pm4a-Pm4h*. Therefore, *PmYAV* might be a new allele at the *Pm4* locus. The identification of *PmYAV* enriched the genetic diversity of *Pm4* locus, which was available for increasing the durable resistance conferred by *Pm4* locus in wheat powdery mildew resistance breeding since it has a resistance spectrum different from that of *Pm4a*, *Pm4b* and *Pm4c*, but unknown with that of *Pm4d* to *Pm4h* which were not tested in the phenotyping study.

Recently, *Pm4b*, which consisted of seven exons and contained two alternative transcripts, was cloned by using the strategy of MutChromSeq (Sánchez-Martín et al., 2021). Subsequently, all the alleles at *Pm4* locus were cloned with the full-length amplification and Sanger sequencing and indicated *Pm4* alleles contain SNPs and/or combinations of shared SNPs which have main effects on the kinase domain. What is even more interesting is that most of the SNP cause amino acid changes in the transmembrane and S_TKc domains. *Pm4* generated two isoforms which were both required for resistance (Sánchez-Martín et al., 2021). In this study, using homology-based cloning, we found that the *Pm4* homologous sequence in YAV249 was consistent with *Pm4a* sequence, while *PmYAV* generated an extra splicing event for the reported *Pm4a_V1* and *Pm4a_V2*, suggesting that there was an extra 67 bp-intron in *PmYAV* (**Figure 4**). It may cause different resistance reactions between *PmYAV* and other alleles. Then, the function of the extra splicing needs to be further investigated in future by other methods, such as virus-induced gene silencing and transgenic assay analysis. On the other hand, Sánchez-Martín et al. (2021) reported that the expression of *Pm4b_V1* and *Pm4b_V2* on the wild-type *Pm4b* wheat genotype Fed-Pm4b after infection with powdery mildew did not significantly differ from each other. Similar expression levels of both transcripts indicate that *Pm4b_V1* and *Pm4b_V2* have a similar contribution to powdery mildew resistance. However, in this study, when inoculated *Bgt* isolate E09, the expression level of *Pm4b_V2* in YAV249 was higher significantly than that of *Pm4b_V1* at most of the time points (**Figure 5**) which might cause the different resistance spectra of *PmYAV* from other alleles at *Pm4* locus.

When a valuable resistance gene or accession was identified, its rational utilization was the first factor to be considered in wheat disease resistance breeding. MAS is a high priority in wheat breeding programs to efficiently transfer *R* gene to susceptible cultivars or pyramid with other *R* gene(s). Sánchez-Martín et al. (2021) reported a dominant diagnostic marker JS717/JS718 which could detect the *Pm4* in different genetic background. In this study, YAV249 showed a high level of resistance to powdery mildew and possessed desirable agronomic traits, such as higher TKW and KL/KW. Two dominant markers WGRC763 closely linked to *PmYAV* and BCD1231 and JS717/718 co-segregated with *PmYAV* and can be used for MAS in many genetic backgrounds. More importantly, the co-dominant marker GDM93 which was closely linked to *PmYAV* could be used for selection of homozygotes during MAS breeding. In fact, an advanced breeding line YK13, which possessed not only desirable agronomic traits but excellent resistance to powdery mildew, was selected in BC_3_F_3_ population of YAV249/KN199 by MAS.

SHW is a valuable genetic resource for improving disease resistance in wheat cultivars. In our study, we identified an extra splicing variant of *Pm4a* from SHW line YAV249 and selected a new wheat breeding line YK13 with high resistance to powdery mildew and excellent agronomic traits. The results will add diversity to the available genes for powdery mildew resistance and provide the foundation for analyzing alternative splicing mechanism in regulating the *Pm4* gene function.

## Conclusion

To sum up, in the present study, we identified a dominant powdery mildew resistance gene *PmYAV* in synthetic hexaploid wheat line YAV249 using bulked segregant analysis with wheat 660K single nucleotide polymorphism (SNP) array scanning and marker analysis. Sequence alignment analysis demonstrated that the sequence of *PmYAV* was totally consistent with that of *Pm4a* but generated an extra splicing. The flanked markers GDM93 and WGRC763, and the co-segregated markers BCD1231 and JS717/JS718 were confirmed to be easily performed in marker-assisted selection (MAS) of *PmYAV*. Using MAS strategy, *PmYAV* was transferred into the commercial cultivar Kenong 199 (KN199) and a wheat line YK13 was selected in BC_3_F_3_ from the population of YAV249/KN199 due to its excellent agronomic traits and resistance to powdery mildew. Our study can be valuable for enhancing the genetic diversity of powdery mildew resistance in breeding.

## Data availability statement

The original contributions presented in the study are included in the article/**Supplementary Material**. Further inquiries can be directed to the corresponding authors.

## Author contributions

HZ and DA conceived the research. YJ, TG, XL, GH and ZS performed the experiments. TG, XL, YZ, JF, JW and HL developed the experimental materials. YZ, JF, JW and WL performed the phenotypic assessment. YJ and TG wrote the manuscript. DA and HZ supervised and revised the writing of the article. All authors contributed to the article and approved the submitted version.

## Funding

This research was supported by the National Key Research and Development Program of China No. 2021YFD1200600, and the National Natural Science Foundation of China No. 32272105.

## Conflict of interest

The authors declare that the research was conducted in the absence of any commercial or financial relationships that could be construed as a potential conflict of interest.

## Publisher’s note

All claims expressed in this article are solely those of the authors and do not necessarily represent those of their affiliated organizations, or those of the publisher, the editors and the reviewers. Any product that may be evaluated in this article, or claim that may be made by its manufacturer, is not guaranteed or endorsed by the publisher.

## Supplementary material

Supplementary Table 1 Validation of the flanked markers GDM93 and WGRC763 and the co-segregated markers BCD1231 and JS717/JS718 of *PmYAV* on 192 wheat accessions in marker-assisted selection (MAS). ‘+’ amplified marker allele differs from that in YAV249, indicating that the marker might be useful in MAS, and ‘−’ marker is similar to that in YAV249, indicating that the marker could not be used in MAS.
